# The Role of miRNAs in Regulating Neurovascular Unit Homeostasis: Bidirectional Communication and Therapeutic Insights in Ischemic Stroke

**DOI:** 10.3390/ijms27031459

**Published:** 2026-02-01

**Authors:** Hongyang Chen, Tianyou Gao, Fengli Ma, Zhuangzhuang Jia

**Affiliations:** 1 School of Basic Medical Sciences, Yunnan University of Chinese Medicine, Kunming 650500, China; chenhyth@163.com (H.C.); jiliezhe@163.com (T.G.); 2 Yunnan Key Laboratory of Integrated Traditional Chinese and Western Medicine for Chronic Disease in Prevention and Treatment, Kunming 650500, China; 3 Key Laboratory of Microcosmic Syndrome Differentiation, Education Department of Yunnan, Kunming 650500, China

**Keywords:** ischemic stroke, miRNA, neurovascular unit, intercellular communication

## Abstract

The neurovascular unit (NVU) is an essential, dynamic multicellular unit that maintains the homeostasis and function of the brain, with the integrity of the NVU having a tremendous impact on the pathogenic progression of ischemic stroke (IS). MicroRNAs (miRNAs) are essential regulators of gene expression and promote intercellular communication and functional unity in the NVU. This narrative review assesses the regulatory process mediated by miRNAs that help maintain homeostasis of the NVU, particularly during IS, with particular emphasis on their modulation of tight-junction (TJ) proteins, basement membrane (BM) and glial–vascular. These regulatory actions are essential for blood–brain barrier (BBB) integrity and neuronal survival. The analysis also exposes the intercommunication networks established by key miRNAs between various cells of the NVU, highlighting their complex and dynamic regulatory properties. Moreover, it investigates therapeutic strategies predicated on miRNA regulatory mechanisms, highlighting the optimistic prospects as well as the present limitations pertaining to effective IS intervention.

## 1. Introduction

The neurovascular unit (NVU) is a concept composed of various cells in the brain and represents a collection of multiple structures and functions of the neurovascular system. It is made of all different cells like the neurons, glia, and blood vessels. The cellular components of the NVU interact with each other through structural and functional interplay in order to maintain the integrity of the BBB and stability of the brain microenvironment, so that these functions can be carried out normally [1,2]. Ischemic stroke (IS) is a common cerebrovascular disease that has a large clinical burden and frequently causes serious dysfunction of the NVU. This functional disorder usually manifests as impaired integrity of the BBB and neuronal damage, which is an important indicator of the deterioration of stroke [3,4,5]. Growing evidence suggests that miRNAs are key non-coding regulators in the complex gene regulatory networks of different cell types within the NVU. And these regulatory factors can not only be regulated to realize the post-transcriptional regulation of the target genes in individual cells, but also communicate with each other outside the cell. In detail, they are carried by exosomes or other extracellular vesicles (EVs) to finally affect the structural or functional state of the NVU [6,7].

IS leads to an ischemic and hypoxic environment, which results in extensive changes in the expression levels of a variety of miRNAs in the brain. These miRNAs will then affect some important disease processes, such as the neuroinflammation process, cell death, glial cell excitation, BBB destruction, and neurorestoration, to form a complex network together [8,9]. Also, miRNAs stabilize the BBB by controlling the production of essential proteins that keep it intact, which either makes or lessens a later ischemic injury to the brain [10]. MiRNAs, due to their multi-targeting nature and importance in inter-cellular signaling, are essential mediators of communications among the different parts of the NVU, which will in turn affect the NVU’s performance [11,12]. Bidirectional information regulation mediated by miRNA not only exists within cells, but is also related to intercellular communication mediated by EVs and other factors. Therefore, targeting specific miRNAs in IS can regulate various intercellular information transmissions within the NVU, indicating broad clinical potential for reconstructing NVU homeostasis and reducing cerebral ischemia-reperfusion injury, providing new options for the treatment of IS [10,13]. This review aims to explore the role of miRNA in maintaining NVU homeostasis, with a focus on miRNA-mediated bidirectional communication between different NVU cells, and introduces the potential and limitations of miRNA for treating IS. These have profound clinical translational significance.

## 2. Regulation of the NVU Structure and Function by miRNAs

**Figure 1:**
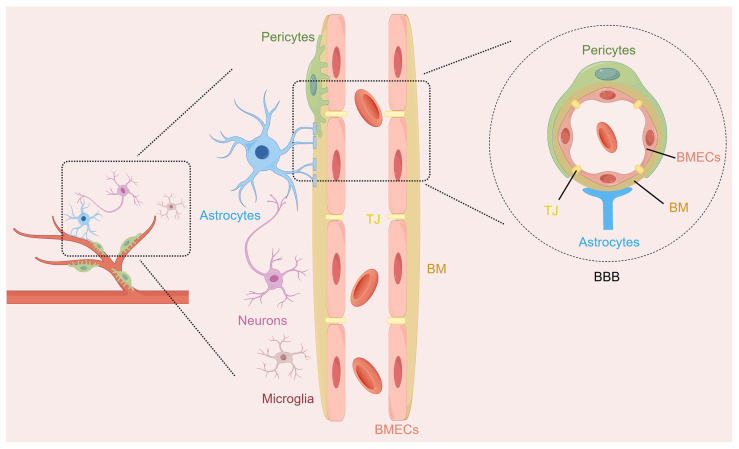
The structure diagram of the neurovascular unit (NVU). The NVU represents a sophisticated structural and functional entity within the brain, comprising multiple cellular components such as neurons, astrocytes, brain microvascular endothelial cells (BMECs), pericytes, and microglia. The blood–brain barrier (BBB), as part of the NVU, contains BMECs and their intercellular tight junctions (TJ), basement membranes (BM), pericytes, and the endfeet of astrocytes.

The NVU is a functional multicellular complex of neurons, astrocytes, brain microvascular endothelial cells (BMECs), pericytes, microglia, and extracellular matrix (Figure 1). The NVU coordinates the activities of its different cell types to regulate cerebral blood flow and maintain BBB integrity, which helps protect brain homeostasis [14]. Neurons are responsible for processing the information in this comprehensive unit and serve as regulatory centers for various signals. Astrocytes, as widely distributed glial cells in the brain, are closely related to neurons, and they establish physical links with the microvessels of the brain through their endfeet. They coordinate neurovascular coupling and promote the clearance of neuronal metabolic waste. BMECs line the inside of the cerebral microvessels and form a kind of filter so that things that are bad for the brain do not enter it from the blood. Pericytes enveloping the periphery of the capillaries can control the tension of the capillaries, thereby maintaining the stability of the BBB. Microglia, as immune regulators in the central nervous system (CNS), focus on the inflammatory cascade within the NVU through physical contact and the release of various substances, such as cytokines, neurocytokines, and angiogenic factors [15,16]. They can realize a bidirectional exchange of information and maintain the stability of the brain microenvironment through such interactions. The structural and functional integrity of the NVU is essential for maintaining the normal functioning of the CNS. The disruption of NVU integrity is strongly linked to the development of numerous neurological diseases. Its dysfunction causes BBB breakdown, starts inflammatory chain reactions, worsens neuron harm in IS, and affects patient results [17,18].

MiRNAs are non-coding endogenous RNA molecules, mostly of length 18–25 nucleotides. They play important roles in controlling basic life processes, including cell proliferation, apoptosis, inflammation, and angiogenesis. They do this regulation by targeting specific mRNAs for post-transcriptional regulation [19,20]. Within the NVU, various cellular populations show different miRNA expression profiles that correspond to their different biological functions. For example, upregulation of miR-424-5p in BMECs may affect the function of microvessels and the permeability of the BBB by inhibiting the fibroblast growth factor 2 (FGF2)/signal transducer and activator of transcription 3 (STAT3) signal pathway [21]. Downregulation of miR-9-5p in astrocytes can promote the proliferation of astrocytes and the secretion of neurotrophic factors, thus promoting nerve repair [22]. MiRNA species of different kinds are contained in exosomes extracted from microglia, and these miRNAs could affect both inflammatory pathways as well as neuroprotective mechanisms [23,24]. At the same time, exosomal miRNAs secreted by pericytes promote angiogenesis and the maintenance of BBB homeostasis [25,26]. In addition, age-related research has shown that the expression of several miRNAs in the BBB changes with age. These miRNAs are implicated in the regulation of vascular tension, the expression of TJ proteins, and inflammatory responses. This highlights their significance for maintaining the NVU and cerebrovascular function [27,28]. MiRNAs’ regulatory effects are not limited to regulating one type of cell internally. In other words, instead of simply influencing the homeostasis and function of each individual cell in the NVU, they operate through intricate intercellular communication networks.

## 3. The Molecular Mechanisms of miRNAs Regulating NVU Homeostasis

### 3.1. MiRNAs in Regulating BBB Integrity

The BBB is a complicated multicellular structure of utmost significance for maintaining brain homoeostasis with the strict control of the bidirectional exchange of blood and the neural parenchyma. Its important structure consists of protein TJ complexes such as occludin, claudin-5, and zonula occludens-1 (ZO-1), which form a continuous sealing band between BMECs [29]. These TJ proteins act like very picky doorkeepers for cells, stopping bad stuff from plasma, poisons, and other not-so-good things from just barging in. Some recent studies show that some miRNAs play crucial roles in the BBB as post-transcriptional regulators. Under normal circumstances, balanced miRNA expression meticulously controls the production and breakdown of TJ components, keeping the barrier in balance. But during cerebral ischemia, there is an imbalance in miRNA regulation, so the up- and downregulation become abnormal. This results in suppression of the expression of critical TJ proteins, a breakdown of the junctional complex, and a decrease in barrier selectivity. Greater paracellular permeability lets blood-borne neurotoxic substances and pro-inflammatory mediators enter the CNS, making secondary neuronal damage and neuro-inflammatory processes in the ischemic penumbra even worse. Therefore, miRNAs play important roles in the maintenance and disruption of NVU and regulate stroke progression.

Following cerebral ischemia, miR-671-5p showed a significant decrease, and its downregulation is linked to impaired BBB integrity. miR-671-5p directly targets a variety of key TJ proteins, such as claudin-5, occludin, and ZO-1, which increases BBB integrity, supports neuron survival, and lessens ischemic damage [30]. Conversely, there are also opposing trends, such as miR-155 being upregulated in inflammatory diseases such as bacterial meningitis and actively participating in BBB disruption. It targets and reduces brain-derived neurotrophic factor (BDNF), leading to fewer TJ proteins like ZO-1, claudin-5, and AQP4. This cascade abnormally boosts BBB permeability and makes the neuroinflammation worse. Inhibiting miR-155 substantially reduces BBB harm and diminishes the discharge of inflammatory mediators, underlining its crucial role in BBB dysfunction during inflammation [31]. Furthermore, miR-142-5p can regulate the degree of damage to the BBB by influencing the level of methionine adenosyltransferase 2B (MAT2B). Inhibiting miR-142-5p promotes the expression of TJ proteins and alleviates inflammation-induced BBB damage [32].

There are different types of cells in the NVU, and miRNAs play a role in determining the mutual crosstalk among various cells in the NVU. A key pathway for this communication includes inflammatory responses like NF-κB and MAPK. For instance, when BMECs are exposed to the dengue virus NS1 protein, abnormal miRNA expression can activate the NF-κB signaling pathway, thus promoting the secretion of pro-inflammatory cytokines, which reduces the expression levels of TJ proteins and results in an abnormal increase in BBB permeability [33]. MiR-466d-3p has been found to be downregulated after exposure to diesel exhaust particles. After intervention with its mimics, it can lead to the downregulation of the Wnt/β-catenin and Wnt/PCP pathways, with decreased protein expressions of Wnt 3 and β-catenin, but increased expressions of TJ proteins ZO-1 and claudin-5 [34]. Moreover, it has been found that miR-27a-3p enhances the expression of claudin-5 and occludin by downregulating glycogen synthase kinase 3 beta (GSK3-β) and upregulating the Wnt/β-catenin pathway, thereby strengthening the stability of the BBB [35]. All of these findings suggest that miRNA can regulate the high or low expression of TJ protein by precisely regulating related signaling pathways, thereby affecting the dynamic homeostasis and pathological repair of the BBB and even the NVU.

### 3.2. Regulation of miRNAs on the Basement Membrane and Glial–Vascular Signaling

The basement membrane (BM) is one of the most important dynamic elements of the NVU, which acts both passively to support the physical structure of cerebral blood vessels and actively as a biochemical messenger, playing a vital role in maintaining the structural and functional integrity of the cerebral blood vessel. This unique type of structure mainly depends on laminin, collagen IV, nidogen, and heparan sulfate proteoglycans, thereby forming a place where endothelial cells within blood vessels, astrocytes in the brain, and pericytes on blood vessels are interconnected. It gives mechanical stability to these constructions, regulates cell connections, and controls the barrier function [36]. More research shows how some miRNAs play important roles in maintaining the structure and function of the NVU. These miRNAs achieve regulatory purposes by precisely controlling the generation, decomposition, and other changes of the important components of the BM. For example, miR-29a is known to be a direct post-transcriptional repressor of a number of collagen genes, such as collagen IV, which is important for inhibiting excessive collagen deposition and thereby ensuring appropriate BM thickness and structural stability. In the diabetic retinopathy model, osteopontin induced a decrease in miR-29a content, which in turn led to abnormal accumulation of collagen IV and pathological thickening of the BM, suggesting that certain miRNAs are involved in the BM generation of the BBB within the NVU [37]. In addition, miR-1298-3p in brain malignant tumors, such as gliomas, exhibits tumor suppressor function by directly binding to and downregulating Nidogen-1, which is a major BM glycoprotein that connects laminin and collagen IV. This decrease hampers the abnormal thickening of the BM, which consequently limits the proliferation and invasiveness of tumor cells [38]. The above results indicate that miRNAs play important regulatory roles in the structural homeostasis of the NVU.

MiRNA is an important signal associated with the glial–vascular system. By influencing communication among astrocytes, endothelial cells, and neurons, it aims to regulate the structure and function of blood vessels and nerves. It is already known that some signaling pathways mediated by endothelial growth factor (VEGF) and transforming growth factor-beta (TGF-β) are very important in angiogenesis and tissue repair. Studies suggest that miRNAs work to regulate the key molecules along these pathways to ensure the functionality of glial cells and blood vessels. For instance, exosomes from adipose tissue-derived mesenchymal stem cells are rich in pro-angiogenic miRNAs, including members of the Let-7 family, the miR-23/27/24 cluster, and miR-1290. These miRNAs promote the repair of damaged blood vessels by stimulating endothelial cell proliferation and neovascularization [39]. Moreover, miRNAs can cause a change in the way in which TGF-β and VEGF signaling works, thus altering the BBB structural and functional features too. And this is based on the fact that the TGF-β and VEGF signaling pathways can modify the complicated interaction between endothelial cells, pericytes, and microglia [40]. Therefore, the finely regulated glial–vascular signaling pathway mediated by miRNA is one of the critical mechanisms for maintaining the homeostasis of the NVU and promoting neuroprotection.

In ischemic conditions, miRNA-mediated BM remodeling is of particular importance. IS leads to structural damage to NVU, thus disturbing the balance of synthesis and degradation of BM components and hindering the recovery of the nervous system. Studies suggest that ischemic states can lead to changes in miRNA expression. These miRNAs are involved in the degradation and reorganization of the BM by regulating related proteins and signal transduction. In this case, through the regulation of the expression of certain miRNAs, one can directly act on their core and regulate the pathways of miRNA degradation and recombination to determine the fate of the BM. For instance, increased miR-7641 levels correspond to too much collagen IV buildup, which causes abnormal thickening of the BM, influencing the blood flow in that spot and showing in a clear way that miRNAs cause pathological remodeling of the BM [41]. MiRNA network regulation, apart from directly acting on the target, can also influence proteases that control the BM. Studies show that miR-3133 affects the expression of matrix metalloproteinase-2 to inhibit the migration of vascular smooth muscle cells and BM degradation, ultimately enhancing vascular stability [42]. These findings collectively reveal the different effects of miRNAs on pathological remodeling or adaptive responses, highlighting their potential as nerve repair agents.

### 3.3. The Regulatory Mechanism of miRNAs on Neuron Survival and Apoptosis

MiRNAs play key roles in regulating neuronal survival and apoptosis signal pathways by post-transcriptional control of gene expression. MiR-21 and miR-124 are two miRNAs that are extensively studied in this context, providing neuroprotection by targeting crucial apoptosis-related genes like B-cell lymphoma 2 (Bcl-2) and different members of the caspase gene family. Experimental models of ischemic brain injury exhibit a reduction in the level of miR-21. Overexpressing miR-21 can inhibit the expression of p53, affect the Bcl-2/Bax signaling pathway, reduce post-ischemic neuronal apoptosis, and promote neuronal survival and the recovery of neural function. This mechanism shows that miR-21 has an anti-apoptotic function in neurons by inhibiting the p53/Bcl-2/Bax signal [43]. Similarly, the expression of miR-124 is also downregulated in the hypoxia–ischemia model. Raising the levels of miR-124 improves neuronal survival, inhibits apoptosis, and improves brain function after hypoxic–ischemic brain injury [44], making it a promising therapy for IS. In addition, another model of neuronal injury was found to have a reduced content of miR-215. After overexpression of miR-215, it was discovered that it could inhibit oxidative stress injury and neuronal apoptosis by targeting large tumor suppressor 2, thereby achieving the purpose of protecting neurons [45].

**Figure 2:**
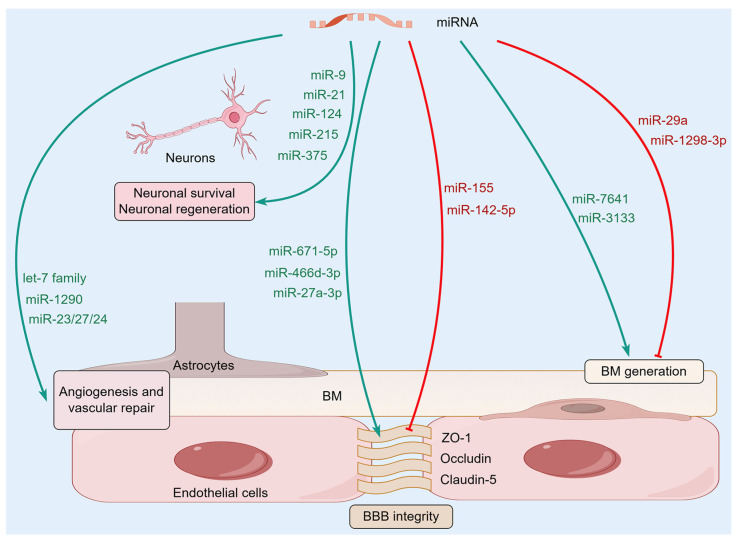
The molecular mechanisms of microRNAs (miRNAs) regulating NVU homeostasis. A variety of miRNAs in the brain can affect the homeostasis of NVU by regulating BBB integrity, BM, and glial-vascular signaling, as well as neuronal survival and regeneration.

In ischemic brain injury, miRNAs have a substantial impact on neuronal regeneration and synaptic remodeling. For instance, miR-375 is activated during the reprogramming process of astrocytes into neurons and thereby participates in neural repair. Overexpressing miR-375 dramatically improves the efficiency of this reprogramming event, greatly improving early neuronal survival and largely maintaining reprogramming neuronal maturation. This selective regulatory action suggests that miR-375 may promote neuronal survival by affecting neuronal ELAVL genes [46]. After intervention with miR-9, cell proliferation was significantly enhanced, and the growth of neurites accelerated, demonstrating a strong promoting effect on neural regeneration. Mechanistically, miR-9 mimic-treated samples exhibited elevated expression levels of Bcl-2 and VEGF, which paralleled the reduced expression levels of pro-inflammatory mediators such as NF-κB1, TNF-α, IL-1β, and iNOS [47]. This molecular mechanism strongly supports the idea that miR-9 has neuroprotective and nerve repair effects after ischemic injury by regulating inflammatory processes and nerve regeneration. In total, all the studies show that miRNAs are engaged in a complex and multiple-target network of regulation with fine control of the survival, apoptosis, and regeneration of neurons. Given the homeostasis regulatory effects of miRNAs on the NVU (Figure 2), they are becoming important molecular mediators of neuroprotective and repair responses in ischemic brain injury, providing promising therapeutic targets for reducing neurological deficits and promoting functional recovery after a stroke.

## 4. The Bidirectional Communication Mechanisms Between NVU Cells Mediated by miRNAs

### 4.1. miRNA Communication Between Neurons and Astrocytes

Neurons carry out intricate intercellular conversations by giving off exosomes loaded with particular miRNAs that afterwards have a considerable regulatory impact on the functional condition of nearby astrocytes. Studies have shown that neuron-derived exosomes carry miR-124-3p to astrocytes for internalization. Once MiR-124-3p enters astrocytes, it produces strong anti-inflammatory results by specifically focusing on and influencing the PI3K/AKT/NF-κB signaling pathway. This molecular intervention can block the pro-inflammatory activation of astrocytes so that too much secretion of inflammatory cytokines and chemokines can be reduced. Therefore, this miRNA-mediated anti-inflammatory response greatly reduces neuronal damage and promotes a favorable environment for neurorepair and regeneration. Also, this transfer of miRNAs between cells inhibits the activation of neurotoxic microglia and astrocytes, which protects damaged neurons from secondary injury following IS [48]. Additionally, neuron-derived miR-181c-5p is involved in astrocytic glutamate uptake, which is essential for NUV homeostasis. It works this way because miR-181c-5p has a precise target to modulate the levels of protein kinase C delta (PKCδ) and glutamate transporter-1 (GLT-1); these two are important proteins for the glutamate transport process. The reduced ability of astrocytes to take up glutamic acid is of great significance, as it can induce excitatory amino acid toxicity [49]. This miRNA-mediated intercellular communication is implicated in the development of multiple neurological disorders, highlighting the importance of neuron–astrocyte crosstalk for the maintenance of the structure and function of the NVU.

Neurons and astrocytes communicating in the NVU are actually part of a very dynamic, bidirectional communication process, not a one-way street. Not only are astrocytes influenced by signals from neurons, but they also regulate the function of neurons through intercellular communication pathways by secreting miRNA-loaded exosomes, thereby affecting the homeostasis of the entire NVU. It is worth noting that exosomes containing miR-382-5p secreted by astrocytes negatively affect mitochondrial function within the neurons after entering them. This process is achieved by targeting and inhibiting the production of optic atrophy 1 (OPA1), an important regulatory factor for mitochondrial dynamics and integrity [50]. Moreover, astrocyte-released exosomes contain a lot of miR-26a-5p, an miRNA that is important for controlling the way dendrites branch out and develop neurons. MiR-26a-5p participates in regulating neuronal dendritic complexity and morphogenesis, thereby influencing the plasticity of neuronal structure and function. This finding shows that astrocytes have the ability to persistently affect neuronal morphology and function by delivering miRNA [51,52]. Moreover, astrocytic EVs carry other neuroprotective miRNAs, such as miR-378a-5p and miR-378b, which display powerful brain protective actions regarding ischemic brain damage by controlling neuroinflammatory reactions and programmed cell death processes [53,54]. All these complicated miRNA communication networks sustain a harmonious two-way conversation between neurons and astrocytes, which is very important for maintaining the integrity of the NVU and helping repair the nervous system after an ischemic event.

### 4.2. MiRNA Signaling Between Neurons and BMECs

There is a very complicated functional synergistic effect between BMECs and neurons that plays an important role in maintaining the structural and functional integrity of the BBB and ensuring the normal physiological functions of neurons. MiRNAs can travel between BMECs and neurons, especially through exosomes. These miRNAs have a great effect on many different biological procedures in the NVU, such as vascular remodeling, BBB integrity preservation, neuron survival, synaptic plasticity, and neuroprotection mechanisms. Specifically, in a cerebral ischemia model, exosomes secreted by BMECs were found to be rich in specific miRNAs, such as miR-27a. This particular miRNA reduces the production of proteins that serve as axonal growth inhibitors in neurons, such as semaphorin 6 A and ras homolog family member A, which effectively promote neural plasticity and regeneration at the molecular level, eventually leading to significant improvement in neural function. This has been proved in previous research [55]. Moreover, EVs from BMECs can pass on miR-146a-5p to neurons. Under cerebral ischemic conditions, miR-146a-5p directly targets and inhibits the expression of Eif4g2, which is one of the eukaryotic translation initiation factors that induces neuron endoplasmic reticulum stress injury [56]. Therefore, it is very likely that miRNAs communicate with neighboring cells through these molecules, which is a major aspect of neuronal survival and regeneration in ischemic brain injury. The influence of neuron-derived miRNAs on BMECs remains unclear, and there are few related studies. However, future in-depth discussions will be crucial to reveal the bidirectional communication between neurons and BMECs.

### 4.3. The Bidirectional Regulation of miRNAs Between Microglia and BMECs

Microglia and BMECs are two of the key cells needed to maintain normal NVU function. They are engaged in miRNA-mediated bidirectional communication and play important roles in regulating the permeability of the BBB and inflammatory reactions. Studies show that when microglia are activated in oxygen-glucose deprivation (OGD), the amount of miR-424-5p encapsulated and released by the exosomes of microglia also significantly increases. This particular miRNA targets FGF2 and mediates the STAT3 signaling pathway. This aggravates BMEC injury and enhances vascular permeability. On the contrary, suppression of miR-424-5p expression effectively alleviates OGD-induced endothelial cell damage, which can restore the ability of these cells to undergo angiogenesis and reduce the permeability of vessels. These results show that miR-424-5p plays an important role in microglia’s negative influence on the structure and activity of BMECs [21]. One more point to note is that miR-124-3p also shows a high expression level in microglia, which promotes neural repair after brain injury. Upon contact with BMECs, miR-124-3p targets the mTOR signaling pathway and inhibits excessive activation of the pathway. This inhibition induces a chain of good changes in BMECs: it promotes autophagy in BMECs, a significant form of programmed cell death that aids in removing damaged or unnecessary parts of cells to maintain equilibrium, and also lowers apoptosis, thus maintaining BMEC quantities and vigor. Beyond this, miR-124-3p upregulates TJ proteins like ZO-1 and occludin. As their expression increases, miR-124-3p helps improve BBB integrity and reduce its permeability. These regulatory mechanisms are quite important for ischemic brain injury [57]. These miRNAs released from microglia significantly affect the integrity of the BBB and the stability of the NVU through intercellular communication pathways, suggesting their potential as therapeutic targets for neurological diseases.

At the same time, BMECs participate in complex feedback regulation with microglia through a complicated miRNA interaction, affecting the inflammatory status of microglia as an important immune effector cell in the NVU. This interaction creates a dynamic two-way conversation network, which is essential to maintain the balance of the neurovascular in both normal and pathological conditions. In particular, miR-3613-3p is a major active secreted miRNA from BMECs and is transported to microglia via exosomes. When it enters microglia, miR-3613-3p specifically matches the 3′ untranslated region of ring finger and CCCH-type domains 1 (RC3H1), a protein that is characteristic of immunoregulation. This binding event results in a substantial decrease in RC3H1 expression levels within microglia. Downregulation of RC3H1 has a significant effect on microglial phenotypic switch. It promotes M1 polarization of microglia, an inflammatory type of microglia characterized by the production of inflammatory cytokines and reactive oxygen species, which can cause neuronal death and impair neuronal function. It is worth noting that after the expression of miR-3613-3p is interfered with in BMECs, it leads to a reduction of miR-3613-3p in BMEC-derived exosomes, which will drive microglia toward M2 polarization [58]. M2 polarization has beneficial effects as it is an anti-inflammatory phenotype of microglia and has a protective effect on nerve tissue from inflammatory damage. In IS and various other neurological diseases, switching the functional phenotype of microglia can affect their immune regulation of the neural environment, thereby regulating the inflammatory response and maintaining homeostasis the BBB. Communication between these BMECs and microglia via miRNAs has a significant impact on the immunomodulatory function of the neurovascular microenvironment.

### 4.4. The miRNA Signaling Network Between Pericytes and Endothelial Cells

In the complicated microenvironment of the NVU, pericytes and BMECs form and maintain a close, highly dynamic, bidirectional communication network. This interaction is carried out via a series of intricate and varied signaling pathways that are precisely regulated to guarantee the normal performance of the NVU. Among the various regulatory molecules that are involved in this bidirectional regulatory process, miRNAs emerge as major players with very fine regulation of gene expression and cell functions. As a type of vascular wall cell surrounding BMECs, pericytes not only provide structural support for blood vessels but also actively participate in the regulatory processes of many physiological activities. They can regulate vascular contraction, control blood perfusion, assist in angiogenesis, and maintain the integrity of the BBB. These effects are crucial for promoting the repair of the NVU affected by ischemic injury. Pericytes release microvesicles containing a specific miRNA as carrier, allowing the transfer of the miRNA to neighboring pericytes [59,60]. Microvesicles derived from pericytes containing miR-145 and miR-132 can be delivered to BMECs and vascular smooth muscle cells. Inside those recipient cells, they can then precisely control how blood vessels behave. In particular, miR-145 promotes the contractile response of vascular smooth muscle cells by activating the Sphk2/S1PR1/MLC20 pathway. Another critical miRNA playing a role in bidirectional regulation is miR-132, which enhances endothelial barrier function through Sphk2/S1PR2/ZO-1 and VE-cadherin signaling [60]. This synergistic effect improves the structural integrity and functional homeostasis of blood vessels.

Another aspect of the mutual influence also needs to be noted; that is, miRNAs from BMCEs of NVU also have huge impacts on pericytes. In a mouse model of middle cerebral artery occlusion and reperfusion, miRNAs released from endothelial cell-derived exosomes, such as miR-126, miR-214, and miR-218, were found to have the capability of improving pericyte proliferation and migration. This is done by regulating the HIF-α/VEGF/DLL4/Notch1 signaling pathway, an important route in angiogenesis, the process of new blood vessel formation, maturation, and stability, leading to greatly improved perfusion of the ischemic areas essential for the survival and recovery of neural tissue after ischemic injury [61]. Moreover, among the endothelial cell-specific miRNAs, miR-126-3p has a distinct regulatory effect on cerebral vascular function by targeting complex angiogenesis and inflammatory pathway genes such as VEGFA, VCAM1, and LPAR2. This coordination not only maintains vascular homeostasis under normal conditions but also supports the repair of injured vessels after an ischemic stroke, contributing to the repair of neural tissue and functional recovery [62].

### 4.5. The miRNA Regulatory Network Between Astrocytes and BMECs

Astroglial endfeet tightly envelop BMECs to strengthen barrier function, which can help with the expression and precise localization of TJ proteins between BMECs. Astrocytes and BMECs cooperate closely to maintain the structure and normal function of the BBB and to regulate cerebral blood flow and neurovascular coupling [63,64]. Exosomes released by astrocytes have particular miRNAs that can be taken up by BMECs and impact their function. For instance, exosomes secreted by OGD-pretreated astrocytes contain a large amount of miR-27a-3p, which can be transported to BMECs and regulate the ARHGAP25/Wnt/β-catenin signaling pathway. This regulation contributes to lesser BBB damage and helps neurological functions recover [65]. Moreover, miR-143-3p released by astrocytes can also enter BMECs. High expression of miR-143-3p inhibits the autophagy-mediated degradation of cell adhesion molecules (CAMs) by targeting the autophagy degradation-related protein ATP6V1A, which results in an increase of CAMs. This makes it possible for neutrophils to cross the BBB and affects inflammatory responses [66]. Selective delivery of miR-143-3p inhibitors to BMECs displayed promising therapeutic results for acute brain injury. Together, these findings show that astrocyte-derived miRNAs have the potential to be used as therapy for vascular barrier repair via exosomes.

Similarly, BMECs of the NVU have the capacity to secrete miRNA-enriched exosomes. These miRNA-carrying exosomes then go on to provide complex feedback regulation to astrocyte functions, which are essential for keeping the NVU stable under both normal and abnormal conditions. Research shows that exosomes released by BMECs are particularly packed with miR-155-5p. This specific miRNA has a different kind of inhibitory influence on the glial-related inflammatory reaction during cerebral ischemia–reperfusion damage, and it accomplishes this regulation by influencing the c-Fos/AP-1 signaling pathway. By interfering with this signaling pathway, miR-155-5p can effectively suppress the inflammatory response in astrocytes and reduce the release of pro-inflammatory cytokines [67]. In addition, exosomes secreted by BMECs are not only miRNAs but also include proteins such as growth differentiation factor 15. These components in exosomes play important roles in activating pro-inflammatory astrocytes, which can cause intense inflammation, thereby intensifying the pathological process inside the brain [68]. Astrocytes and BMECs use this bidirectional communication method through miRNA-carrying exosomes to dynamically control the NVU microenvironment, which is very important for maintaining balance in the tiny blood vessels in the brain so that they can work normally.

### 4.6. The Synergistic Effect of miRNA Signaling Between Glial Cells

Glial cells are abundant, functionally diverse groups of cells found in the CNS. Among them, astrocytes and microglia communicate through the miRNA-mediated signaling pathway, especially via EVs carrying miRNAs, regulating the inflammatory response and neuro-reparative mechanisms. For example, after a traumatic brain injury, microglia release miR-142, which activates astrocytes and promotes the transformation of astrocytes into an inflammatory phenotype that strongly upregulates genes like pro-inflammatory ones (IL-1β, PTGS2), leading to increased inflammation propagation linked to brain injury [69]. Moreover, the study shows that miR-145-5p carried by microglia exosomes can bind to Smad3 mRNA and regulate Smad3 protein levels via a post-transcriptional mechanism. This specific interaction decreases the amount of Smad3 protein in astrocytes, which stops astrocytes from growing and prevents scarring by astrocytes after injury to the spinal cord. These findings indicate that miR-145-5p acts as a negative regulator of astrocyte proliferation. Downregulation of miR-145-5p promotes the activation of Smad3 and astrocyte proliferation [70].

**Figure 3:**
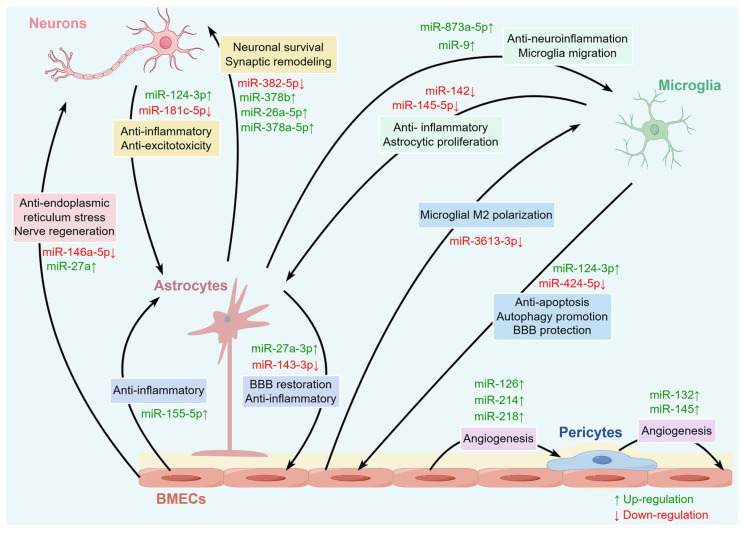
The miRNAs involved in various intercellular communications within the NVU. A variety of miRNAs in the brain can regulate intercellular communication among various cells within the NVU, including neurons, BMECs, microglia, astrocytes, and pericytes.

Meanwhile, EVs released from astrocytes are abundant in different miRNAs, which in turn affect microglia. Research has shown that miR-873a-5p, a major component of astrocyte EVs, substantially hampers the lipopolysaccharide-induced shift towards the M1 phenotype of microglia by inhibiting the NF-κB signaling pathway, which reduces microglia-mediated neuroinflammation and improves neurological deficits after traumatic brain injury [71]. Additionally, when microglia come into contact with exosomes derived from astrocytes loaded with miR-9, the migration level of microglia intensifies. This situation occurs because miR-9 downregulates the phosphatase and tensin homolog (PTEN). This highlights the role of miRNAs in glial intracellular communication [72]. Crucially, miRNAs drive signal transduction between glial cells, which has expanded from simple inflammation control to comprehensive effects on neuroprotection and regeneration. Modulating the metabolic state of glia, programmed death, expression of neurogenic factors, recruitment and differentiation of neural stem cells driven by miRNA signals, and increasing neurogenesis contribute to the reconstruction of neural networks [73,74]. Specifically, miRNAs can regulate processes such as immune inflammation, programmed cell death, and neural remodeling through multiple signaling pathways, participating in interactions among various cells within the NVU (Table 1). From the perspective of the multi-cell coordination mentioned above, miRNA-mediated intercellular communication plays a crucial role in maintaining the structural and functional homeostasis of the NVU (Figure 3).

## 5. New Strategies and Translational Prospects for IS Treatment Based on miRNA Regulatory Mechanisms

### 5.1. MiRNA Mimics and Inhibitors in the Regulation of NVU

MiRNA-targeted therapy could restore NVU function and improve the pathological conditions in IS by controlling key miRNAs. During pathological processes, miRNA mimics or inhibitors are employed to precisely regulate abnormal miRNA expression. The miRNA mimics or inhibitors are capable of controlling crucial elements in IS, such as inflammation, cell death, and BBB stability. MiRNA mimics are artificially made copies of real miRNAs, copying the functions of natural miRNAs and helping control target genes. Inhibitors, as antisense oligonucleotides, bind to and block target miRNAs, preventing them from functioning biologically [75]. These tools can help in regulating NVU balance and repairing ischemic brain tissue. For example, the levels of miRNA-29a-5p and miRNA-126a-5p decrease after ischemia. Using mimics to restore their protective effects can reduce damage to the BBB and the death of neurons while maintaining NVU balance. On the contrary, inhibitors could block the abnormally increased miRNAs, such as miRNA-195-3p expression, to reduce inflammation and oxidative stress after ischemia/reperfusion and prevent them from damaging the neurovascular system [9]. Additionally, the non-coding RNA network shows the interactions between miRNAs, circRNAs, and lncRNAs, which regulate the fate of NVU cells under ischemia/reperfusion. Utilizing agonists and inhibitors to modify this network allows for exact interventions concerning brain injury and functional deficits. For example, circRNA affects miRNA activity and thus influences mRNA expression, forming circRNA–miRNA–mRNA axes that help in neuroprotection and vascular repair. Designing corresponding agonists and inhibitors for these axes will lead to new ways of precisely treating IS [11].

However, unmodified synthetic miRNA molecules are easily captured by the host immune system, leading to low delivery efficiency and potentially adverse immune responses. To address this bottleneck, modern delivery platforms innovatively introduce chemical modification techniques, nanocarriers, and natural carriers such as exosomes. These strategies not only effectively avoid immune surveillance but also significantly enhance the stability and brain-targeting ability of miRNA, greatly improving the safety and feasibility of treatment. Nanocarriers and EVs are currently the main carriers of miRNA delivery in the field of delivery technology. Among them, synthetic nanocarriers, due to their controllable size and surface modification capabilities, can target miRNA delivery to brain injury sites [76,77], becoming one of the mainstream carriers for miRNA delivery. However, they may induce host immune responses, leading to inflammatory responses and tissue toxicity, which can affect treatment efficacy and increase the risk of adverse reactions. To address immunogenicity issues, surface modification has become one of the key strategies to reduce immune responses. By modifying the carrier surface with polyethylene glycol or coating it with natural biological membrane components, the recognition and clearance of the carrier by the immune system can be effectively reduced, extending its blood circulation time and improving delivery efficiency. At the same time, surface modification can also improve the biocompatibility of the carrier and reduce inflammatory responses, thereby enhancing overall safety [78,79,80]. EVs are another type of carrier for miRNA delivery. Due to their natural biocompatibility, lower immunogenicity, and potential to cross the BBB, they are gradually becoming a promising delivery platform [81,82]. Compared with traditional synthetic nanoparticles, these natural carriers show superior advantages in reducing immune activation and inflammatory responses. However, their biologically active components are complex, and extraction and purification techniques are still immature. How to systematically evaluate their immunogenicity and ensure their therapeutic stability is an urgent issue that needs to be addressed. The composite delivery system, as an innovative strategy in miRNA delivery technology, aims to combine the advantages of nanoparticles and EVs, utilizing the loading and targeting capabilities of nanoparticles while leveraging the biocompatibility and immune-regulating functions of EVs, significantly enhancing delivery efficiency and safety [83,84]. Although the composite delivery system shows significant advantages theoretically, its design complexity and high manufacturing process requirements limit the pace of clinical translation. Future research needs to continue to delve into aspects such as preparation process optimization, functional validation, and safety assessment to promote the transition from laboratory to clinical application.

### 5.2. Progress in Preclinical and Clinical Research

In recent years, a lot of progress has been made in miRNA research on IS treatment, including fundamental research on the mechanism, preclinical animal model experiments, and clinical trial attempts. MiRNAs are involved in regulating gene expression in different cell types of NVU, affecting BBB integrity, inflammation, apoptosis, and repair. Thus, they represent interesting therapeutic targets for IS [8,11,85]. In animal models, miRNA-regulated intercellular communication in the NVU directly impacts pathological changes after IS. Let-7 family miRNA, such as let-7g*, and miR-98, can obviously decrease the production of pro-inflammatory cytokines in IS models and suppress inflammation, improve neurovascular perfusion, and promote neuroprotection [86]. In addition, downregulated miR-155-5p in peripheral blood mononuclear cell secretion after OGD is associated with the upregulation of VEGF and stem cell markers. The secretome modifies the glial microenvironment, supports angiogenesis and axonal growth, and helps in the recovery of function after a stroke [87]. Together, these outcomes give very strong proof that miRNAs play an important role in communication between many different cells in the NVU, which creates a good basis for future strategies to help patients with cerebral infarction.

The safety and efficiency of miRNA-centered interventions for stroke patients are getting a lot of interest in the medical world. According to current research, miRNAs can serve as an important therapeutic target and as an indicator for diagnosis and prognosis. Moreover, particular miRNAs display gender-specific expression patterns that could lead to different stroke pathophysiologies and responses to treatment between genders [88,89,90]. Moreover, miRNAs derived from the NVU and packaged in exosomes exist in the circulatory system, which implies that they might become biomarkers for early diagnosis and treatment monitoring [91]. It is worth mentioning that in recent years, the therapeutic strategy of EVs derived from stem cells carrying miRNA has gradually gained attention in clinical trials. The results of a prospective randomized controlled trial showed that after mesenchymal stem cell therapy, the EVs in the bloodstream of patients with IS significantly increased, and the miRNA related to neurogenesis carried by them (such as miR-18a-5p) also significantly increased, indicating the potential of miRNAs carried by EVs in mediating the improvement of neurological deficits and promoting neural repair in patients with IS [92].

Nevertheless, current clinical trials on miRNA therapy for IS are still in their infancy, and there are no miRNA drugs approved for clinical use. Related research is mostly focused on preclinical studies, and clinical translation still faces many challenges. The first issue is the off-target effects of miRNA. MiRNAs can target multiple mRNA molecules, leading to non-specific gene regulation. Although this characteristic endows miRNAs with the ability to regulate complex signaling networks in biological systems, it may also cause a series of side effects, affecting the clinical efficacy and long-term health of patients, becoming one of the key issues that need to be addressed in miRNA therapy applications. Currently, strategies to reduce off-target effects mainly include modification of miRNA molecules and optimization of delivery systems, combined with computational biology methods for the prediction and screening of high-specificity miRNAs to achieve more precise targeted therapy. Among these, miRNA modification techniques are one of the key means to reduce off-target effects. Through structural modifications, such as chemical modifications or binding with specific ligands, the stability and targeting of miRNA can be enhanced, reducing its expression in non-target cells or non-target areas. For example, studies have used α-tocopherol as a delivery ligand to construct heteroduplex oligonucleotides of miR-126 inhibitors, which are efficiently delivered to the BMECs in the ischemic brain area through intravenous injection, significantly inhibiting the expression of miR-126, and the delivery efficiency in the ischemic brain area is significantly higher than that in the non-ischemic brain area, indicating that such modification and delivery strategies can effectively improve targeting and reduce off-target effects [93]. Secondly, there are also many challenges in miRNA delivery. In addition to immunogenicity issues, the pharmacokinetic problems of miRNA delivery significantly restrict its clinical application in IS, especially the barriers to crossing the BBB, targeted delivery to specific cells within the NVU, the stability of the delivery system in the blood, and its release kinetics remain key bottlenecks limiting its clinical translation. Currently, related research mostly stays at the in vitro experimental or preliminary animal model stage, lacking systematic and dynamic in vivo pharmacokinetic data, especially the delivery efficiency and release behavior targeting specific cell types within the complex NVU structure that have not been fully elucidated. Therefore, future research needs to integrate multidisciplinary technical means, especially combining advanced imaging technologies (such as multimodal in vivo imaging) and precise biomarker tracking, to monitor in real time the distribution, metabolism, and miRNA release process of the delivery system in vivo, thereby more accurately assessing the pharmacokinetic performance of the delivery strategy. In addition, there is another important issue: most clinical trials mainly focus on short-term efficacy assessments, making it difficult to comprehensively reflect the adverse reactions or potential risks that miRNA therapy may bring in the long term. Related studies suggest that some miRNAs are upregulated during vascular injury repair, such as miR-21-3p, miR-29-3p, and miR-26a, which are increased under conditions of vascular stenosis and may be related to vascular wall injury and repair processes. However, whether the long-term overexpression of these miRNAs will lead to adverse gene expression disorders or promote pathological processes (such as excessive vascular proliferation, fibrosis, etc.) remains unclear [94]. Establishing a scientifically reasonable long-term follow-up and safety monitoring system is a key link to achieving its clinical translation. Therefore, future efforts should focus on solving the challenges of miRNA targeting, delivery efficiency, stability, and long-term safety to promote its clinical application in patients with IS and achieve the clinical translation of miRNA therapeutic strategies.

### 5.3. Application Prospects of miRNA as a Precise Biomarker

MiRNAs have emerged as precise molecular biomarkers with tremendous potential for the early diagnosis and prognosis of IS. They are constantly found in the body’s easily accessible fluids like blood and cerebrospinal fluid, which makes them easy to get without hurting the person, making them really good signs of what is going on inside [95,96]. The microRNA expression profile in blood and cerebrospinal fluid has been found to be related to stroke pathogenesis. Numerous miRNAs have been found to be involved in expression alterations at an early stage of stroke, according to previous studies. For instance, miR-494 is greatly upregulated in the lymphocytes of patients who have had a stroke, and it is strongly linked to good outcomes in younger patients [97]. Large-scale plasma miRNA analyses also identified miRNAs like miR-7-5p, miR-140-5p, and miR-210-3p as significantly differentially expressed between different time points of IS, which are potential dynamic markers for IS [98]. In particular, miRNAs encapsulated in exosomes may have higher diagnostic accuracy. They are able to more accurately reflect pathological changes in the brain and can cross the BBB, thus making them better stroke biomarkers [6,99]. MiRNAs are showing increasing promise as diagnostics and prognostics for IS. Their particular expression patterns closely match the early stroke severity and the later functional recovery, better than traditional biomarkers, which just provide static disease information. Using plasma miR-129-1-3p as an illustration, its levels are closely connected to the degree of neurological deficits, forecasting useful restoration in stroke patients, and it also helps differentiate between different stroke subtypes and guides subtype-specific therapies [100]. Also, miR-155 expression levels in plasma endothelial microvesicles are positively associated with the volume of cerebral infarction and neurological deficit scores. Combining the detection of its expression levels and the counts of endothelial microvesicles greatly improves diagnostic accuracy [101]. A certain study has also shown that the expression level changes of the miR-34a/SIRT1 axis are closely related to the pathological severity of IS, suggesting that this signaling pathway can be used as a predictor of disease severity [102]. The results indicate that miRNA could serve as indicators reflecting the condition of stroke pathologic processes, and it also has the ability to track and predict patient recovery by the progression of the disease.

In addition, based on clinical characteristics and imaging examinations, IS can be classified into different subtypes, mainly including large artery atherosclerotic stroke, cardioembolic stroke, and small artery occlusion. These subtypes exhibit significant differences in pathogenesis and clinical manifestations; therefore, their molecular markers, especially the expression profiles of miRNAs, also show uniqueness. For example, the polymorphism of miR-300 is significantly associated with susceptibility to IS, particularly large artery atherosclerotic stroke, while the polymorphisms of miR-10a and miR-34b/c are related to the mortality of stroke patients [103]. The polymorphism of the binding site of miR-181a with its target gene MTMR3 shows a significant association in large artery and small artery atherosclerotic stroke subtypes, and this polymorphism is also related to the severity of stroke and short-term prognosis, further supporting the role of miRNA expression and regulation in the diagnosis of stroke subtypes [104]. In addition, circRNA, as a “sponge” for miRNA, also participates in the pathological process of stroke subtypes by regulating miRNA activity. Studies have found that certain circRNAs are significantly differentially expressed in large artery atherosclerotic and cardioembolic strokes and are closely associated with the miRNA regulatory network, which further enhances the role of miRNA as diagnostic markers for stroke subtypes [105]. Moreover, it is worth noting that miRNAs also play a key role in regulating the efficacy and safety of thrombolytic therapy for IS. For example, the levels of miR-335 and miR-224 are significantly correlated with the severity of IS and infarct volume, and miR-335 shows high diagnostic accuracy in predicting adverse outcomes for patients, indicating that they not only reflect the severity of brain tissue damage, but may also be involved in the pathological mechanisms of hemorrhagic transformation after thrombolysis [106]. miR-411-5p is also significantly upregulated in patients with acute cerebral infarction after thrombolysis and is closely related to NIHSS scores and cerebral perfusion parameters (cerebral blood flow and cerebral blood volume). When used in conjunction with computed tomography perfusion parameters, it can accurately predict short-term and long-term prognosis after thrombolytic therapy, demonstrating its practical value as a biomarker for assessing the efficacy and prognosis of thrombolytic therapy in IS [107].

Although miRNA biomarkers have many potential clinical application prospects, there are still many obstacles to their clinical application. Considering their usually low concentrations in vivo, developing highly sensitive detection techniques is necessary. Furthermore, different studies may have methodological differences, leading to inconsistent results [108,109]. Based on the above considerations, it is necessary to develop a detection platform with higher sensitivity and specificity. New technologies, such as enzyme-free amplification strategies based on DNA tetrahedral nanostructures, have greatly improved the ability to find miRNAs, which is helpful for quantifying miRNAs with extremely low concentrations in plasma, implying great potential for miRNAs in early clinical diagnosis [110]. In addition, there is a lack of unified standardized processing in the preparation procedures for biological samples, miRNA separation parameters, and specific operation rules for other detections, which hinders the repeatability of research data from one study to another [109,111]. To get past these limitations, establishing uniform technical criteria and conducting multicenter trials are necessary. As a multi-target regulatory factor, the inherent biological complexity of miRNA has exacerbated the predicament of its clinical transformation. To improve the use of miRNAs in diagnosing and predicting clinical outcomes, it is urgent to integrate the data on clinical parameters and other bio-omics [112,113].

## 6. Discussion

As a result of gaining an understanding of the complex structure and function of the NVU, miRNAs have been noted to be important intercellular signaling molecules in investigating the pathology of IS. Much evidence indicates that certain miRNAs have different regulatory impacts on BBB integrity, neuronal health, and glial activity, demonstrating their complex regulatory features. In addition to improving the understanding of how NVU homeostasis is disturbed, miRNA-mediated intercellular communication is important for ischemia. Current research has greatly advanced the understating of how miRNAs impact inflammatory pathways, BBB permeability, and repair through fine-tuning of NVU cell communication. Though there are different miRNA targets and mechanisms as a result of different experimental models, samples, and detection methods, the general finding is that they play a positive role in the repair of the BBB, the protection of neurons, and the remodeling of neurovasculature. In this context, the use of systems biology and multi-omics integration is a powerful way to reconcile the different results found in the research and to better understand miRNA regulatory networks in IS. Moreover, therapeutic methods utilizing miRNA regulation mechanisms have clear benefits in terms of multi-target synergistic regulation. They may at the same time impact many vital areas of the NVU, laying out a new direction for precise remedies for IS.

Although existing data show that miRNA therapy has good safety and efficacy, this field still faces multiple challenges, urgently requiring a scientific and reasonable balance of different research perspectives and findings to promote its healthy development. It is important to note that some miRNAs, such as miR-155, exhibit a complex dual effect in IS, promoting inflammation response and BBB damage while also inhibiting excessive inflammation through negative feedback mechanisms, thus playing a neuroprotective role. The key variables that may affect the differential effects of miRNA include multiple aspects. The first issue is that there are differences in the time points of the analysis. The expression of miRNA after IS shows significant time dependence, with distinct differences in miRNA expression profiles and functions during the early inflammatory phase and the later repair phase. The regulation of miRNA at different time points not only reflects the dynamic changes of the pathological process but also affects its diagnostic and predictive value as a biological marker. Future research should comprehensively consider time factors, precise sampling, and analysis to more accurately reveal the mechanisms of miRNAs in IS, providing reliable evidence for clinical diagnosis and treatment. Furthermore, different dosages of intervention methods will also have different effects. The dosage of miRNA has a decisive influence on the selective regulation of target genes and the activation of signaling pathways. Different concentrations of miRNAs may activate entirely different intracellular signaling networks, thereby regulating various key processes, such as cell survival, apoptosis, and inflammation response. This also suggests that precise control of dosage is crucial when designing miRNA-related interventions. Ignoring this can easily lead to unstable efficacy or even adverse reactions. Another point worth noting is the influence caused by differences in the genetic background and age of animals. Different strains of animals exhibit significant differences in miRNA expression profiles and immune responses, which directly affect the pathological processes mediated by miRNAs. Animal models with different genetic backgrounds show different miRNA regulatory patterns in the inflammatory response, apoptosis, and neurorepair mechanisms after IS, indicating that genetic diversity may lead to differences in miRNA function. Age is another key factor affecting miRNA function. With increasing age, gene expression profiles undergo significant changes, especially in the expression of genes related to immune inflammation, with older animals showing stronger inflammatory responses and immune cell activation. This further emphasizes the need to consider the comprehensive impact of genetic background and age on miRNA function in animal model studies and clinical intervention designs to enhance the precision and effectiveness of IS treatment strategies. Similarly, the existence of metabolic diseases should not be ignored. Metabolic diseases such as diabetes mellitus and hypertension are important risk factors for IS and significantly affect the expression and function of miRNAs, thereby regulating the pathological process of IS. Metabolic abnormalities typically alter the inflammatory environment in the body, regulating the expression profiles of miRNAs, which may amplify or inhibit the neuroprotective or pathogenic effects of certain miRNAs and become key factors influencing the prognosis of IS. Therefore, metabolic status, as an important background factor affecting miRNA function, should be included in the stratified analysis design of IS-related miRNA research to improve the specificity and clinical translational value of these studies. The fact that ischemic experimental models, detection methods, and other factors also influence the expression characteristics of miRNA should also be taken seriously. Currently, commonly used models of ischemic brain injury mainly include the transient middle cerebral artery occlusion model, embolic model, and photothrombotic model. Each of these models has its characteristics. The transient middle cerebral artery occlusion model is widely used in ischemia-reperfusion injury research due to its simulation of vascular occlusion and reperfusion processes, while the embolic model is closer to the pathological state of clinical thrombosis, and the photothrombotic model induces local thrombosis through photosensitizers, suitable for exploring microvascular injury mechanisms. However, these models exhibit significant differences in ischemic duration, blood flow recovery, and inflammatory responses, leading to considerable variations in miRNA expression profiles and NVU responses and affecting the comparability of research results and their clinical applicability. One more point is that existing miRNA detection methods, such as microarrays, RNA-seq, and qPCR, each have their own characteristics, but there are significant differences in sensitivity, specificity, and artifact generation, affecting the accuracy and reliability of detection results. At the same time, the lack of a unified reference gene standard makes it difficult to directly compare data between different laboratories, limiting the promotion and clinical application of miRNA research.

Overall, the field of miRNA therapy for IS is in a stage of rapid development, with broad prospects, but still facing severe challenges. Future research should pay more attention to multidisciplinary team collaboration, optimize molecular design and delivery technologies, create a more complete and reliable miRNA detection platform, improve the safety assessment system, establish more representative stroke models, systematically analyze the spatiotemporal expression characteristics and functional mechanisms of miRNAs in different NVU cells, and conduct multicenter, large-sample clinical studies to verify treatment outcomes and safety. Only by ensuring specificity and safety can the deep clinical translation of miRNA therapy be achieved, thereby truly benefiting a large number of IS patients.

## Figures and Tables

**Table 1:** miRNA-mediated bidirectional communication within the NVU.

Donor Cell	Recipient Cell	miRNA	Target	Function	References
Neurons	Astrocytes	miR-124-3p	PI3K/AKT/NF-κB signaling pathway	Inhibition of neurotoxic astrocyte activation, inhibition of A1-type astrocyte activation, reduction of neuroinflammatory response	[48]
		miR-181c-5p	PKCδ, GLT-1	Reduces the ability of astrocytes to uptake glutamate, leading to synaptic excitatory neurotransmission dysfunction	[49]
Astrocytes	Neurons	miR-382-5p	OPA1	Neuronal mitochondrial damage and neurological dysfunction	[50]
		miR-26a-5p	Neural cell adhesion molecule axis, AKT/GSK3-β/CRMP2 signaling pathway	Dendritic complexity and morphological development regulation of neurons, improved dendritic development, and reduced neuronal apoptosis	[51,52]
		miR-378a-5p miR-378b	Pyroptosis-related proteins Gasdermin D, NLRP3, Caspase-1 p20	Inhibition of neuroinflammation and programmed cell death	[53,54]
BMECs	Neurons	miR-27a	Semaphorin 6A, Ras Homolog Family Member A	Axonal remodeling, nerve regeneration	[55]
		miR-146a-5p	Eif4g2	Promotes endoplasmic reticulum stress injury in neurons	[56]
Microglia	BMECs	miR-424-5p	FGF2/STAT3 pathway	Promotes damage to BMECs and increased vascular permeability	[21]
		miR-124-3p	mTOR signaling, ZO-1, Occludin, Beclin-1, p62, LC3-II/LC3-I	Promotes autophagy of BMECs, reduced cell apoptosis, improves BBB integrity, reduced BBB permeability	[57]
BMECs	Microglia	miR-3613-3p	RC3H1	Promotes polarization of microglia M1 and reduces neuronal survival	[58]
Pericytes	Endothelial cells	miR-132	Sphk2/S1PR2/ZO-1 and VE-cadherin signaling pathway	Improves the barrier function of vascular endothelial cells	[60]
		miR-145	Sphk2/S1PR1/MLC20 signaling pathway	Enhances vasoconstriction	[60]
Endothelial cells	Pericytes	miR-126 miR-214 miR-218	HIFα-VEGF-DLL4-Notch1 signaling pathway	Promotes the proliferation and migration of pericytes, improved the maturation and stability of neovascularization	[61]
Astrocytes	BMECs	miR-27a-3p	ARHGAP25/Wnt/β-catenin signaling pathway	Reduces the damage to the BBB and promotes the recovery of neurological function	[65]
		miR-143-3p	ATP6V1A, CAMs	Promotes CAM expression, guides neutrophil migration across BMECs into the brain, exacerbates inflammatory response	[66]
BMECs	Astrocytes	miR-155-5p	c-Fos/AP-1 signaling pathway	Inhibition of inflammatory response and apoptosis within astrocytes, promotion of cell proliferation, improvement of neurological deficits	[67]
Microglia	Astrocytes	miR-142	IL-1β, PTGS2	Enhances the pro-inflammatory state of astrocytes and exacerbates the spread of inflammation	[69]
		miR-145-5p	Smad3	Inhibition of astrocyte proliferation	[70]
Astrocytes	Microglia	miR-873a-5p	NF-κB signaling pathway	Inhibits the transition of microglia to M1 phenotype and alleviates microglial-mediated neuroinflammation	[71]
		miR-9	PTEN	Promotion of migration of microglia	[72]

PI3K—Phosphoinositide 3-kinase, AKT—Protein kinase B, NF-κB—Nuclear factor kappa B, PKCδ—Protein kinase C delta, GLT-1—Glutamate transporter-1, OPA1—Optic atrophy 1, GSK3-β—Glycogen synthase kinase 3 beta, CRMP2—Collapsin response mediator protein 2, NLRP3—NOD-like receptor protein 3, Eif4g2—Eukaryotic initiation factor 4 gamma 2, FGF2—Fibroblast growth factor 2, STAT3—Signal transducer and activator of transcription 3, ZO-1—Zonula occludens-1, RC3H1—Ring finger and CCCH-type domains 1, Sphk2—Sphingosine kinase 2, S1PR—Sphingosine 1 phosphate receptor, MLC20—Myosin light chain 20 kDa, HIFα—Hypoxia-inducible factor alpha, VEGF—Vascular endothelial growth factor, DLL4—Delta-like ligand 4, ARHGAP25—A Rho GTPase-activating protein 25, CAMs—Cell adhesion molecules, AP-1—Activating protein 1, IL-1β—Interleukin-1 beta, PTGS2—Prostaglandin-endoperoxide synthase 2, Smad3—Small mother against decapentaplegic family member 3, PTEN—Phosphatase and tensin homolog.

## Data Availability

No new data were created or analyzed in this study. Data sharing is not applicable to this article.

## Abbreviations

The following abbreviations are used in this manuscript:

NVU	Neurovascular unit
IS	Ischemic stroke
miRNA	MicroRNA
TJ	Tight junction
BM	Basement membrane
BBB	Blood–brain barrier
EVs	Extracellular vesicles
BMEC	Brain microvascular endothelial cell
CNS	Central nervous system
FGF2	Fibroblast growth factor 2
STAT3	Signal transducer and activator of transcription 3
ZO-1	Zonula occludens-1
TJAP-1	Tight-junction-associated protein 1
BDNF	Brain-derived neurotrophic factor
MAT2B	Methionine adenosyltransferase 2B
GSK3β	Glycogen synthase kinase 3 beta
VEGF	Vascular endothelial growth factor
TGF-β	Transforming growth factor-beta
Bcl-2	B-cell lymphoma-2
PKCδ	Protein kinase C delta
GLT-1	Glutamate transporter-1
OPA1	Optic atrophy 1
OGD	Oxygen-glucose deprivation
RC3H1	CCCH-type domains 1
CAMs	Cell adhesion molecules
PTEN	Phosphatase and tensin homolog
